# Pilot Investigation of Blood Pressure Control Using a Mobile App (Cardi.Health): Retrospective Chart Review Study

**DOI:** 10.2196/48454

**Published:** 2023-10-17

**Authors:** Marius Nakrys, Sarunas Valinskas, Kasparas Aleknavicius, Justinas Jonusas

**Affiliations:** 1 Kilo.Health Vilnius Lithuania; 2 Lithuania Business University of Applied Sciences Klaipėda Lithuania

**Keywords:** mobile app, Cardi.Health, blood pressure, engagement, app, pilot study, hypertension, effective, blood pressure control, self-monitoring, medication, management, engagement, users, use

## Abstract

**Background:**

The high prevalence of hypertension necessitates effective, scalable interventions for blood pressure (BP) control. Self-monitoring has shown improved adherence to medication and better BP management. Mobile apps offer a promising approach with their increasing popularity and potential for large-scale implementation. Studies have demonstrated associations between mobile app interventions and lowered BP, yet real-world data on app effectiveness and engagement remain limited.

**Objective:**

In this study, we analyzed real-world user data from the Cardi.Health mobile app, which is aimed at helping its users monitor and control their BP. Our goal was to find out whether there is an association between the use of the mobile app and a decrease in BP. Additionally, the study explored how engagement with the app may influence this outcome.

**Methods:**

This was a retrospective chart review study. The initial study population comprised 4407 Cardi.Health users who began using the app between January 2022 and April 2022. After applying inclusion criteria, the final study cohort comprised 339 users with elevated BP at the baseline. The sample consisted of 108 (31.9%) men and 231 (68.1%) women (*P*=.04). This retrospective chart review study obtained permission from the Biomedical Research Alliance of New York Institutional Review Board (June 2022, registration ID 22-08-503-939).

**Results:**

The study’s main findings were that there is a possible relationship between use of the Cardi.Health mobile app and a decrease in systolic BP. Additionally, there was a significant association between active use of the app and systolic BP decrease (*χ*^2^_1_=5.311; *P*=.02). Finally, active users had an almost 2 times greater chance of reducing systolic BP by 5 mm Hg or more over 4 weeks (odds ratio 1.932, 95% CI 1.074-3.528; *P*=.03).

**Conclusions:**

This study shows a possible relationship between Cardi.Health mobile app use and decreased BP. Additionally, engagement with the app may be related to better results—active use was associated with an almost 2-fold increase in the odds of reducing BP by 5 or more mm Hg.

## Introduction

The prevalence and burden of hypertension are exceptionally high. It is estimated that in the United States, nearly half of all adults have hypertension, and of those, only approximately 1 in 5 achieve adequate control of their blood pressure (BP) [1]. Elevated BP is one of the most significant risk factors for ischemic heart disease and stroke and thus is a leading preventable cause of cardiovascular disease and heart failure [2-4]. Accordingly, reducing the prevalence of hypertension is a worldwide target for preventing and controlling noncommunicable diseases [5].

Despite the many strategies available for hypertension management, sufficient blood pressure control in the population is evidently lacking. Thus, there is a growing need to establish and test scalable interventions to achieve said control [6,7]. In addition, research has noted that self-monitoring of BP leads to better control and improves adherence to medication [8-10]. One recognized way of promoting the self-management of BP is mobile apps [11]. It is thought that by providing an organized overview of BP data, information about hypertension, the correct ways to measure BP, and relevant lifestyle modifications, mobile apps may make it easier for patients to understand and adequately control their BP [12]. Importantly, considering the growing popularity of mobile phones and health apps [12], mobile technology interventions are a way of disseminating information that may potentially be scaled for large populations.

Existing studies demonstrate an association between mobile app interventions and lowered BP. For example, in a large cohort study, engagement in a BP self-management program that used a mobile app with automated lifestyle coaching was associated with lower BP within a follow-up period of 3 years [6]. Similarly, systematic reviews of mobile app BP interventions note that most controlled studies report associations with lowered BP [13-15]. Nevertheless, there is still relatively little real-world data about the effectiveness of and engagement with mobile BP apps. More research about the features, mechanisms of BP control, engagement factors, and effectiveness of mobile app interventions targeting BP in various settings and populations is still required.

In this study, we aim to analyze real-world user data of the Cardi.Health mobile app, with the specific objectives of determining whether there is an association between the use of the mobile app and a decrease in BP and how engagement with the app may influence this outcome. We hypothesize that increased engagement with the Cardi.Health mobile app will be associated with a significant decrease in blood pressure among its users. We hope to provide insight into the effectiveness of mobile interventions in real-world settings, thus adding to the growing body of knowledge about this promising field of mobile health.

## Methods

### Ethical Considerations

This retrospective chart review study received research ethics approval from the Biomedical Research Alliance of New York Institutional Review Board in June 2022 (22-08-503-939). The need for patient consent was waived due to the retrospective nature of the investigation. The study was conducted according to the guidelines of the Declaration of Helsinki.

### Participants

The initial study population comprised 4407 Cardi.Health users who began using the app between January 2022 and April 2022. Users who met the following criteria were included for further investigation: they entered their age, gender, height, and starting weight; they had at least one active day per week with valid app-related activities (eg, they documented activities, entered data, completed an exercise from the app, or looked for nutrition information in the app) for 4 consecutive weeks; they entered blood pressure measurement results at least once a week; and they had less than 30 active days overall. After applying the inclusion criteria, the final study cohort comprised 339 users with elevated BP at the baseline. The sample consisted of 108 (31.9%) men and 231 (68.1%) women (*P*=.04). Detailed descriptive data are presented in Table 1.

**Table 1:** Descriptive data of the final cohort (n=339).

		Men		Women		*P* values
Users, n (%)		108 (31.9)		231 (68.1)		.04
**Users in starting blood pressure category, n (%)**						
	Elevated	39 (31.2)	86 (68.8)		.29	
	High	33 (27.5)	87 (72.5)		.72	
	Very high	36 (38.3)	58 (61.7)		.77	
Active days, median (IQR)		23 (11)		21 (9)		.42
Total time of use (days), median (IQR)		47 (59)		43 (54)		.63

### Intervention

**Figure 1:**
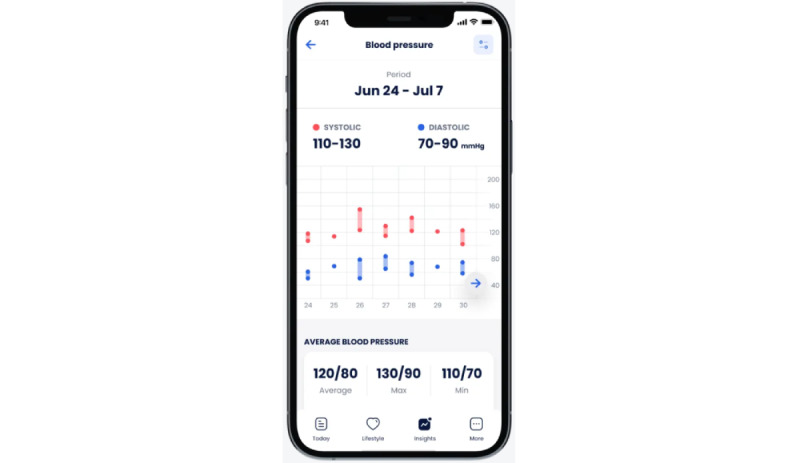
Cardi.Health mobile app screen for symptom logging.

**Figure 2:**
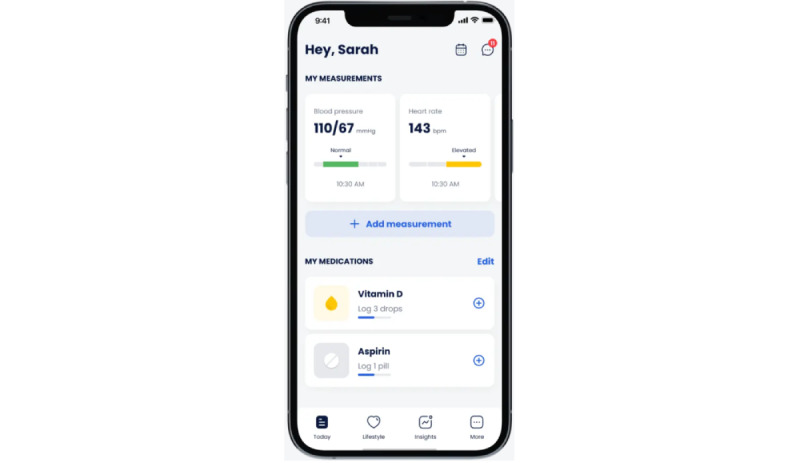
Cardi.Health mobile app screen for medication logging.

**Figure 3:**
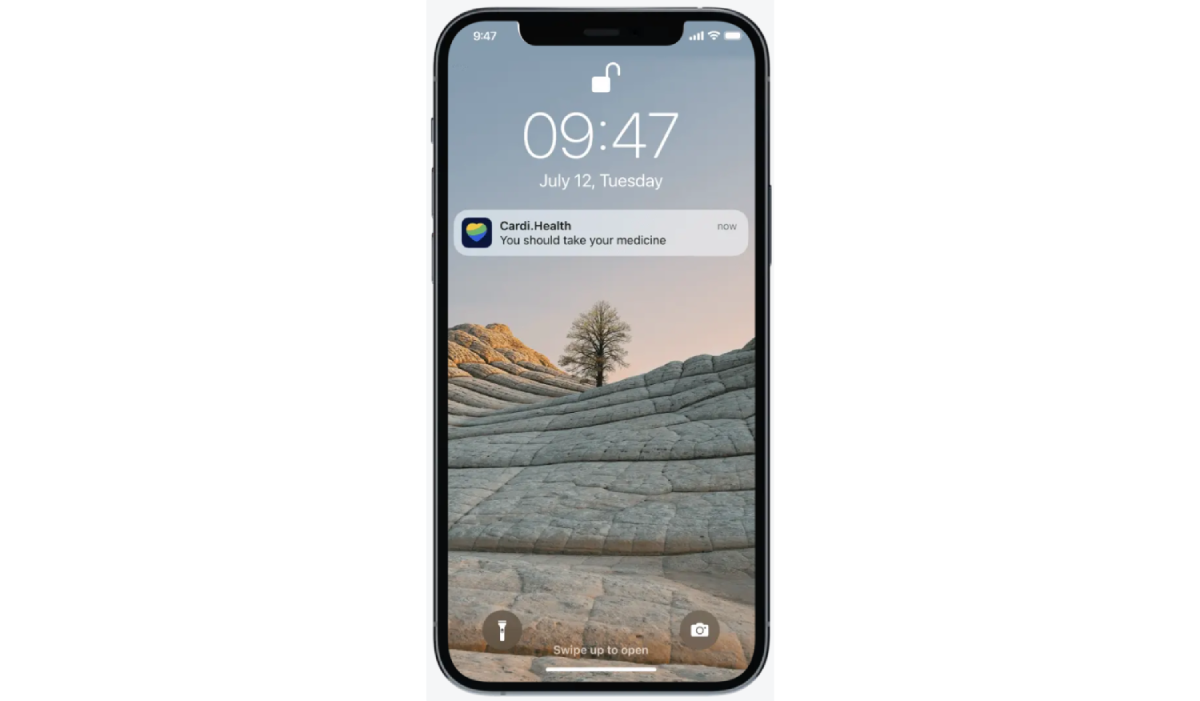
Cardi.Health mobile app giving a reminder.

**Figure 4:**
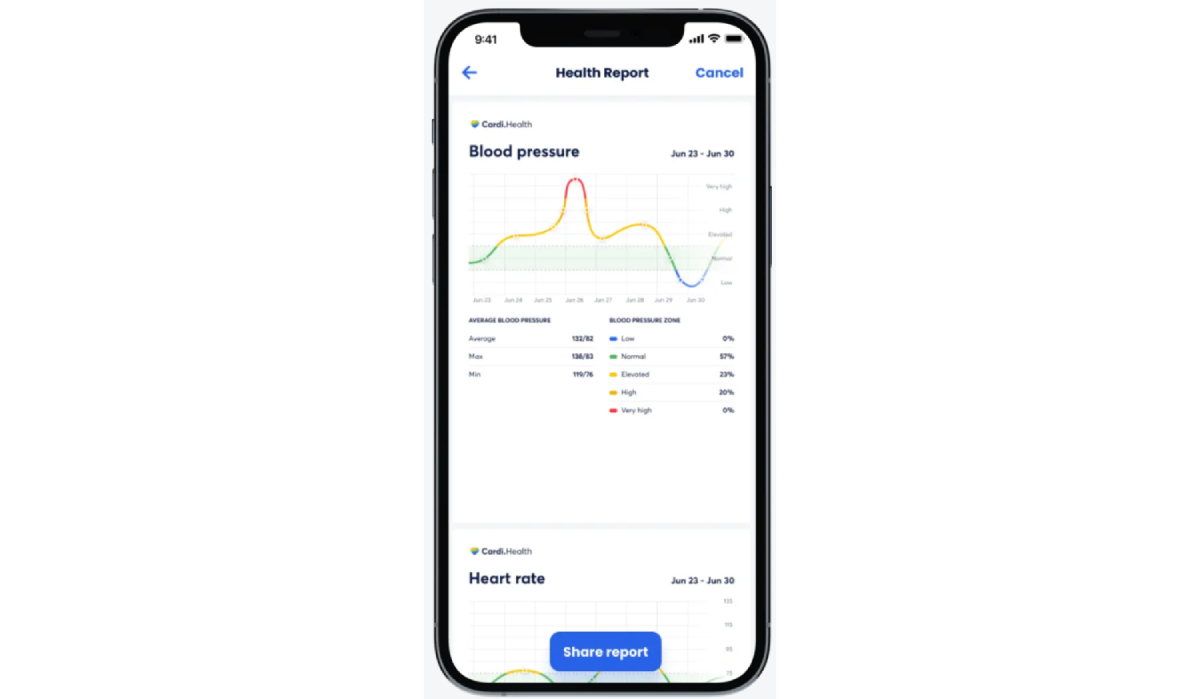
Generated report for a scheduled physician check-up.

Cardi.Health is available for download from the App Store and the Google Play store. The app serves as a tool for the daily monitoring and management of cardiovascular diseases. It enables users to track their condition by receiving regular reminders to check their vital signs (recommendations and instructions about the proper techniques for self-measurements, such as BP, are also provided) and to log their symptoms (Figure 1) and medications (Figure 2), and it provides actionable insights and reminders (Figure 3). In addition, the app features personalized insights, condition trackers, daily reminders for medication, insight action plans, and meal and activity plans, and, finally, generates reports for a scheduled physician check-up (Figure 4). The app was developed in collaboration with cardiologists working clinically and the American Heart Association’s Center for Health Technology and Innovation.

### Measures

Systolic BP values were used to assess the effectiveness of the app in managing users’ BP levels over a consecutive 4-week period. Users were classified into 3 groups based on their mean systolic BP values during their first week of app use: elevated (120-129 mm Hg), high (130-139 mm Hg), and very high (140 mm Hg or higher) [16].

To assess user engagement over the 4-week period, an “activity ratio” was calculated. This ratio was obtained by dividing the number of active days (ADs) by the total time (TT) the app was used. Both ADs and TT are measured in days. The activity ratio ranged from 0.04 to 1, with a score of 1 indicating that the user had the app for x number of days and was active and engaged with the app on all x number of days. The activity ratio allowed the identification of users who frequently used the app during the 4-week period. Furthermore, it enabled comparing 2 user groups based on their activity ratio: users who frequently used the app during the 4-week period and those who used it for 4 weeks but were less active.

### Statistical Analyses

The statistical analysis of the data was performed using GraphPad Prism (version 9; GraphPad Software). The normality of continuous variables was assessed using the Shapiro-Wilk test. For related nonnormally distributed samples, the Wilcoxon signed-rank test was used to analyze paired or unpaired samples. A chi-squared analysis was used to investigate the relationship between activity ratio and users who lowered their systolic BP by 5 mm Hg or more over 4 weeks. The sample was divided into 2 groups based on activity ratio: active (activity ratio ≥0.75) and nonactive (activity ratio <0.75). The frequency distribution of users who achieved the systolic BP reduction threshold was calculated for each group. A significance level of .05 was chosen for all analyses.

Furthermore, a logistic regression model was used to examine significant differences between mean systolic BP values during the first and fourth weeks of app use. The change in systolic BP values was selected as the dependent variable and coded as follows: users whose systolic BP decreased by 5 mm Hg or more over 4 weeks were coded as 1, while those whose systolic BP decreased by less than 5 mm Hg or increased were coded as 0. The covariates included the users’ activity level, categorized as nonactive or active based on the activity ratio (where an activity ratio of 0.75 or higher indicated an active user). A logistic regression model was constructed to investigate the effect of the independent variables (covariates) on the dependent variable.

## Results

The statistical analysis using the Wilcoxon signed-rank test showed that both groups significantly reduced systolic BP within the elevated, high, and very high BP categories. It is worth mentioning that active users did not experience a statistically significant change in systolic BP in the elevated BP category (Table 2). However, we observed a tendency toward decreased BP (*P*=.09).

The chi-squared test results (*χ*^2^_1_=5.311; *P*=.02) indicate a significant association between activity and systolic BP decrease. The active group had a higher proportion of participants who decreased systolic BP over 4 weeks by more than 5 mm Hg than the nonactive group.

Finally, based on the activity ratio, a logistic regression model was created to predict the likelihood of reducing systolic BP by 5 mm Hg or more over 4 weeks. The model showed that users with an activity ratio equal to or more than 0.75 had an almost 2 times greater chance of reducing systolic BP by 5 mm Hg or more over 4 weeks than users with an activity ratio less than 0.75 (odds ratio [OR] 1.932, 95% CI 1.074-3.528; Table 3).

**Table 2:** Systolic blood pressure results at baseline and 4 after weeks of use of the app.

Blood pressure category		Systolic blood pressure (mm Hg), mean (SD)			*P* value
		Week 1	Week 4	Difference	
**Elevated**					
	Nonactive (n=107)	125.6 (2.88)	124.6 (9.58)	–1.06 (9.18)	.02
	Active (n=18)	125.1 (3.21)	121.9 (7.85)	–3.18 (7.70)	.09
**High**					
	Nonactive (n=93)	134.5 (2.87)	129.7 (11.32)	–4.79 (11.44)	<.001
	Active (n=27)	134.5 (2.64)	127.1 (8.28)	–7.42 (8.71)	<.001
**Very high**					
	Nonactive (n=81)	150.2 (10.33)	140.3 (14.22)	–9.91 (15.73)	<.001
	Active (n=13)	146.2 (6.46)	134.0 (12.21)	–12.17 (12.22)	.006

**Table 3:** Logistic regression model predicting the likelihood of reducing systolic blood pressure by 5 mm Hg or more.

Variable	Coefficient	SE	*P* value	Odds ratio (95% CI)
Nonactive vs active	0.652	0.302	.03	1.932 (1.074 to 3.528)

## Discussion

### Principal Findings

The primary finding of this study is a potential association between the use of the Cardi.Health mobile app and a reduction in systolic BP. Across all participant groups, there was a discernible trend toward decreased BP associated with app use. Notably, among active users, engagement with the app was linked to a decrease in systolic BP of over 5 mm Hg during the 4-week period. Furthermore, the logistic regression model indicated that active users had nearly double the likelihood of experiencing a decrease in BP of 5 mm Hg or more (OR 1.932, 95% CI 1.074-3.528).

The use of mobile apps in the field of BP control has become increasingly popular in recent years. These apps provide a convenient and accessible way for individuals to monitor their BP levels on a regular basis. A recent randomized control trial (RCT) conducted by Gazit et al [6] showed promising results for further use of such apps. The use of a mobile app designed for improved BP control led to reductions in mean systolic BP of 7.2 (SE 0.4) mm Hg, 12.2 (SE 0.7) mm Hg, and 20.9 (SE 1.7) mm Hg in groups with elevated BP, stage 1 hypertension, and stage 2 hypertension, respectively, in comparison with baseline measurements. Additionally, the authors found that engagement with the app was associated with lower BP (131.2, 133.4, and 135.5 mm Hg for low-, medium-, and high-engagement groups, respectively; *P*<.05). On the other hand, in an RCT by Persell et al [17], there was no statistically significant difference between intervention and control groups when comparing BP differences after 6 months of intervention (–2 mm Hg, 95% CI –4.9 to 0.8; *P*=.16). However, the intervention group’s self-confidence in controlling BP was statistically significantly higher (0.4 points on a 5-point scale, 95% CI 0.2-0.5; *P*<.001).

Our results show a similar trend and a possible relationship between app use and a decrease in BP. Users in all BP groups (except for the active elevated BP group, where the tendency was observed but was not statistically significant) statistically significantly lowered their BP during the time they used the app. Such results may be explained by the fact that mobile apps increase adherence to medication and diet, which was shown in a study by Bozorgi et al [18], who reported that mobile intervention increased treatment adherence by 5.9 points (95% CI 5.03-6.69) based on the Hill-Bone scale. Similar conclusions were drawn by 2 systematic reviews, whose authors found that mobile apps tend to increase adherence to medications and subsequently decrease BP [19,20].

It is worth mentioning that more engaged use was associated with a higher chance of reducing BP by 5 mm Hg or more in our study. We speculate that a few effects may be in play to explain such an observation. First, a mobile app for BP control can help increase awareness of one’s BP levels, leading to better control [13]. Additionally, by allowing users to track and monitor their BP levels over time, mobile apps can offer users valuable insights into how their BP responds to various activities and medications throughout the day [21]. This can empower users to make informed decisions about their lifestyle choices and medication use, ultimately improving their ability to manage their BP. Finally, dedicated BP control apps can provide users with educational resources and support [22]. These resources can include tips for managing BP and information about lifestyle changes.

### Limitations

As with all similar investigations, the retrospective nature of this study design may introduce the possibility of recall bias, where participants may not accurately remember or record all relevant information in the mobile app. Secondly, the study may be subject to selection bias, as the data were obtained from a self-selected group of mobile app users. This may limit the generalizability of the findings, as the study sample may not be representative of the broader population of individuals with high BP. Additionally, a significant limitation of our study is the requirement of payment to use the app. This creates a potential health equity issue, as it may inadvertently exclude individuals from lower socioeconomic backgrounds, who may not be able to afford the app. This is particularly concerning given that hypertension and other noncommunicable diseases disproportionately affect individuals from lower socioeconomic backgrounds. Therefore, our findings primarily represent individuals who can afford the app and may not be generalizable to the broader population. This limitation underscores the importance of considering affordability and accessibility when developing and implementing digital health interventions. Future research and intervention development should prioritize inclusivity and explore alternative funding models, such as subsidies or free versions with in-app purchases, to ensure that mobile health apps are accessible to a broader audience, thereby addressing health equity more thoroughly. Moreover, as the information we could obtain about the app users was limited, we faced challenges in establishing causality between mobile app use and BP control, as the data may not account for other factors that could influence BP control, such as medication adherence, lifestyle habits, and comorbidities. Furthermore, we are unable to confirm whether the users were using a clinically validated BP device. Most BP devices available on the market are not validated for clinical accuracy, which raises concerns about the accuracy and reliability of the BP data entered by the users. While the app provides recommendations and instructions about the proper techniques for self-measurements of BP, it does not have the capability to confirm the accuracy or validation status of the devices used by the users. This limitation raises the possibility that some of the BP data entered into the app may not be clinically accurate, which could potentially impact the findings and conclusions of this study. Finally, a significant limitation is the frequency of BP measurements, as the users were only required to enter their BP data at least once per week. This is inconsistent with clinical guidelines for self-monitoring of BP, which recommend a minimum of 3 days per week of BP measurements, and ideally, daily measurements to calculate the average for assessing significant BP changes or control [23]. This discrepancy may affect the accuracy and clinical relevance of the BP data collected and analyzed in our study.

### Conclusions

Despite the increasing popularity of mobile app interventions and the increasing number of studies analyzing the results such apps generate, their results should be interpreted with a grain of salt. Considering all possible limitations, this study shows a possible relationship between Cardi.Health mobile app use and decreased BP. Additionally, engagement with the app may be related to better results—active users were associated with an almost 2-fold increase in the odds of reducing their BP by 5 or more mm Hg. An RCT will be initiated to test these results and provide more robust conclusions on the efficacy of the Cardi.Health app.

## Abbreviations

AD: active day
BP: blood pressure
OR: odds ratio
RCT: randomized control trial
TT: total time

## Data Availability

The data sets generated and analyzed during this study are available from the corresponding author upon reasonable request.
